# Virtual Reality: A New Frontier of Physical Rehabilitation

**DOI:** 10.3390/s25103080

**Published:** 2025-05-13

**Authors:** Alessandro Capriotti, Sarah Moret, Eleonora Del Bello, Ario Federici, Francesco Lucertini

**Affiliations:** Department of Biomolecular Sciences, University of Urbino Carlo Bo, 61029 Urbino, Italy; s.moret@campus.uniurb.it (S.M.); e.delbello@campus.uniurb.it (E.D.B.); ario.federici@uniurb.it (A.F.); francesco.lucertini@uniurb.it (F.L.)

**Keywords:** immersive virtual reality, physical rehabilitation, upper limb, adult, elder

## Abstract

Immersive virtual reality (VR) technology has enhanced the field of physical rehabilitation by offering a novel approach to motor recovery and serving as an effective assessment tool. It enables the simulation of various actions, including activities of daily living, within immersive, safe, and controlled environments. Although numerous studies have examined the efficacy of immersive VR for upper limb motor recovery in patients with various conditions, this review aimed to summarize current evidence, highlight benefits and limitations, and provide directions for future research. The review was conducted following PRISMA guidelines. Studies involving individuals over 18 years old with stroke, Parkinson’s disease, amputation, or fibromyalgia were included. The findings demonstrate improvements in strength, dexterity, range of motion, and coordination. Additional benefits included enhanced neuroplasticity and pain reduction. Immersive VR-based exercise sessions were often perceived as more enjoyable and engaging, and less complex, than conventional therapy. The technology proved to be safe, adaptable across age groups, and customizable. Furthermore, the integration of immersive VR into telerehabilitation programs improved accessibility for both patients and healthcare providers. However, not all populations may benefit equally from this method due to variability in disease severity and the presence of comorbidities.

## 1. Introduction

Immersive virtual reality (IVR) is an emerging technology garnering increasing interest within the research community due to its wide range of applications, particularly in the cognitive–motor domain. This innovative approach involves human–computer interaction within augmented or immersive VR settings, enabling users to respond realistically to virtual stimuli [1]. Recent advancements and the development of cutting-edge technologies have significantly contributed to progress in physical rehabilitation. These innovations offer novel approaches to motor recovery and represent valuable assessment tools capable of detecting outcome measures with high accuracy and objectivity [1,2,3]. A key feature of VR is the creation of an immersive environment that simulates both everyday and uncommon activities through multisensory stimulation in a safe and controlled setting. This immersive quality enhances patient engagement and is often perceived as both motivational and entertaining. Additionally, VR environments can be tailored to the residual capabilities of individual users [1,3].

Proprioception provides the central nervous system with real-time information about biomechanical parameters, such as speed, force, direction, and acceleration, as well as physiological changes in muscles, tendons, and joints. However, visual illusions can alter proprioceptive perception. In rehabilitation, such illusions are frequently employed to enhance therapeutic outcomes. For instance, Bourdin et al. used IVR to manipulate visual feedback regarding arm position, thereby improving motor performance [4]. The sense of embodiment in IVR arises when individuals observe a virtual body that closely resembles their own, with realism and first-person perspective serving as critical factors for eliciting ownership of the virtual body [5].

Over the past few decades, various tools have been developed to enhance upper limb rehabilitation [6]. Among them, VR has proven valuable due to its ability to provide real-time feedback via sensors, thereby improving both motor performance and learning. It also positively influences patient motivation [7]. Furthermore, VR interventions are adaptable to different age groups and individual needs. Multiple studies have reported that IVR-based rehabilitation sessions are perceived as less painful, more enjoyable, and more engaging compared to conventional therapies. This increased engagement helps reduce perceived discomfort and boredom during therapy [8,9,10].

VR has been employed in the rehabilitation of a wide range of motor impairments, particularly those resulting from neurological conditions such as Parkinson’s disease (PD), multiple sclerosis (MS), cerebral palsy, and stroke [2]. Studies utilizing this technology have reported significant functional improvements in both preventing deterioration and facilitating recovery. As global life expectancy increases, the burden of neurological disorders on public health continues to grow. In 2016, motor impairments represented one of the leading causes of increased disability-adjusted life years (DALYs), with upper limb dysfunction being particularly prominent [11]. Although IVR has been increasingly studied as a training modality in neurological rehabilitation, the majority of research focuses on post-stroke recovery, with relatively limited evidence available for PD, amputation, and pain-related conditions. Moreover, most studies are centered on the adult population [12].

Stroke is a neurological condition characterized by the death of neurons in specific brain regions due to disrupted blood flow and consequent oxygen deprivation. Symptoms manifest rapidly and may include paralysis, sensory deficits, spasticity, facial asymmetry, language and comprehension difficulties, headaches, balance and vision disturbances, lack of coordination, and loss of consciousness [11]. Approximately 80% of stroke survivors exhibit upper limb impairments [13], yet only one-third regain satisfactory hand function in the chronic recovery phase [14], just 26% resume their daily activities independently [14], and only 26% of them will be able to perform daily activities again [13]. Stroke remains the most common cause of long-term disability in adults and is the second leading cause of dementia globally. This imposes a substantial economic burden on healthcare systems and society at large, contributing to unmet neurorehabilitation goals such as those established in the United States for 2023 [15]. The integration of novel technologies is urgently needed to facilitate skill recovery, reduce rehabilitation costs, enhance accessibility, and decrease the number of therapists required per session. In this context, VR represents a promising telerehabilitation solution [11].

PD is a slowly progressive neurodegenerative disorder primarily affecting dopaminergic neurons in the substantia nigra [16]. PD is associated with a broad spectrum of motor and non-motor symptoms. Non-motor symptoms include chronic pain, fatigue, sleep disturbances, and cognitive and mood alterations [17]. Motor manifestations commonly include resting tremors, bradykinesia, rigidity, and impairments in balance and gait. These affect both gross and fine motor skills, resulting in diminished dexterity and difficulties in performing basic activities of daily living (BADLs) [16]. As there is currently no cure for PD, treatment focuses on symptom management and slowing disease progression. Physical activity plays a crucial role, especially in early-stage management [11]. VR-based interventions have shown efficacy in improving gait, balance, and overall mobility. Emerging research supports the feasibility, safety, and effectiveness of IVR for individuals with PD [18].

In the United States, approximately two million individuals undergo limb amputation each year, and this number is projected to double by 2050. Upper limb amputation results in significant functional limitations, primarily due to the loss of hand grip, which negatively affects BADLs and non-verbal communication. The consequences of surgical amputation extend beyond functional impairment, encompassing both psychological and physical effects, such as phantom limb pain. Myoelectric prostheses are commonly prescribed to mitigate these challenges; however, patients often face long delays before receiving their prosthesis. Additionally, the effective use of these devices requires targeted muscle training, which enables patients to contract specific muscles to optimize prosthesis control. Traditional training methods are frequently perceived as monotonous and lack interactive feedback, often leading to difficulties in prosthesis handling [18]. This prolonged and demanding process often results in patients discontinuing their rehabilitation programs [19]. Various emerging technologies have been employed to reduce dropout rates, with IVR showing particular promise. IVR’s provision of visual feedback significantly enhances the acquisition of control skills, boosts intrinsic motivation, and increases patient engagement and effort during rehabilitation [18].

Chronic pain is among the most frequently reported symptoms by patients in clinical settings. According to the International Association for the Study of Pain (IASP), approximately 20% of the global adult population is affected by chronic pain. This condition has multifaceted consequences, including physical and physiological effects on individuals, as well as broader economic and social impacts. Fibromyalgia Syndrome (FS), for example, is one of the most prevalent causes of chronic pain, with chronic pain itself being the primary symptom of the condition [20]. Physical activity is a key factor in improving quality of life for patients with chronic pain. It contributes to the restoration of lost functionality and has positive effects on mood. However, certain symptoms of FS, such as fatigue and persistent tiredness, often discourage movement. As a result, patients may enter a vicious cycle of ‘pain–inactivity–pain’, which progressively exacerbates their condition [21]. VR may offer a promising solution for managing chronic pain, primarily through its capacity to provide distraction-induced pain relief. Additionally, VR can serve as a supportive tool in facilitating physical activity and promoting functional recovery. Emerging studies and clinical trials have reported encouraging outcomes, suggesting that IVR could become a viable therapeutic option for individuals with FS in the near future [22].

Numerous studies have investigated the effectiveness of IVR in promoting upper limb motor recovery across a range of clinical conditions. This review aims to synthesize existing evidence, highlight the associated benefits and limitations, and offer recommendations to guide future research in this field.

### Objective

This study aims to evaluate the use of IVR as a therapeutic tool for upper limb rehabilitation in individuals with various pathologies, including neurological disorders and fibromyalgia. The study population consisted of adults experiencing upper limb impairments or fibromyalgia symptoms, who underwent supervised rehabilitation protocols incorporating IVR. The analysis focused on improvements in residual motor function and the recovery of lost or impaired motor skills.

## 2. Materials and Methods

This systematic review was performed according to the guidelines in the Preferred Reporting Items for Systematic Review and Meta-Analysis (PRISMA) [23]. The PICOST framework was also employed to formulate the research questions and to guide the design of the systematic review (Table 1).

### 2.1. Eligibility Criteria

A literature search was conducted to evaluate the effectiveness of IVR protocols in individuals with upper limb motor disorders and fibromyalgia. Eligibility criteria included studies involving adult participants diagnosed with upper limb conditions or fibromyalgia and treated with IVR therapy. Pathologies represented by fewer than two published articles were excluded. Included studies comprised randomized–controlled trials (RCTs), analytical studies, and case reports published in English between 2018 and 2023.

### 2.2. Research

**Figure 1:**
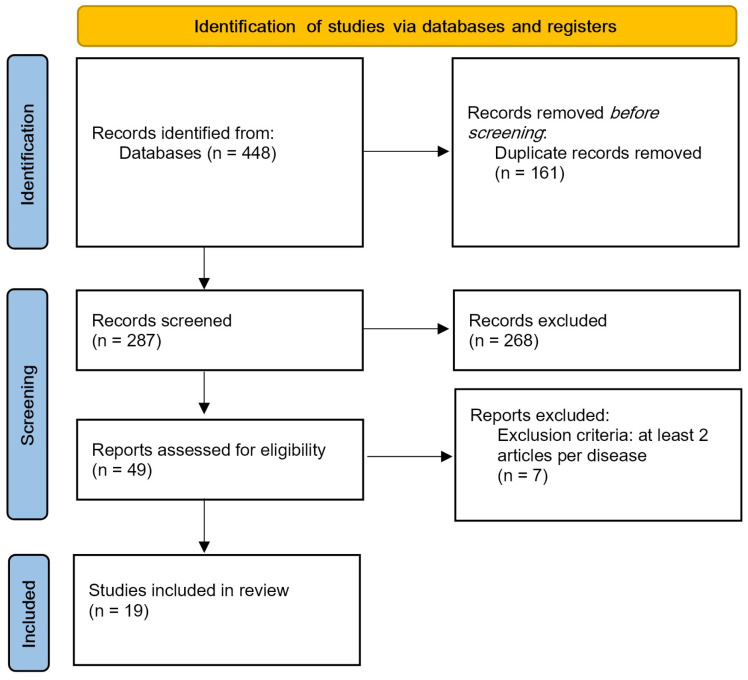
PRISMA flow diagram.

A research strategy for significant literature has been developed for PubMed and adapted for other databases, such as ACM and Web of Science. The keywords used were “immersive virtual reality upper limb” and “immersive virtual reality fibromyalgia”. Filters included publication date (from 2018 to 2023) and article type; 448 articles were analyzed, and 19 articles were included (Figure 1).

### 2.3. Information Sources

All articles retrieved from the databases were imported into Rayyan software to facilitate the screening process. The initial screening involved the automatic removal of duplicate records. The second phase consisted of manually reviewing potential duplicates and excluding articles that did not meet the predefined eligibility criteria. Finally, the remaining articles were categorized by pathology (stroke, Parkinson’s disease, amputation, fibromyalgia) and included only if at least two studies addressed the same condition.

### 2.4. Data Collection

This research was carried out independently. Data regarding participants, interventions, experimental and control groups, results, and limits were extracted from each selected study.

## 3. Results

The results are presented in Table 2, Table 3, Table 4, Table 5 and Table 6, which list the papers grouped by clinical area.

## 4. Discussion

This review examines the application of IVR across a range of medical conditions, including acute and subacute stroke, chronic stroke, PD, limb amputation, and FS. The review highlights the potential benefits and limitations of IVR-based interventions and emphasizes the need for further research. The following sections provide a detailed analysis of each condition.

### 4.1. Acute/Subacute Stroke

The development of upper-limb training protocols incorporating new technologies, such as IVR, is essential to support functional recovery. These protocols should be grounded in motor learning principles. IVR also has the potential to enhance motor skills by promoting neuroplasticity [24]. Studies have demonstrated that supplementing conventional therapy with one additional hour of IVR training leads to greater motor improvement in the affected limb and induces cortical changes, compared to standard physical therapy alone [25]. Another study confirmed that limb mirroring training using IVR promotes neuroplasticity, resulting in motor improvements in the affected limb [24]. Park and collaborators analyzed improvements in ideomotor apraxia in a stroke patient. After 12 weeks of IVR training, the patient showed improvement in nearly all symptoms. These findings suggest that IVR may be an effective tool for the rehabilitation of ideomotor apraxia [26]. The main limitations identified include small sample sizes, short intervention periods, and a limited body of literature available for reference [24,25,26].

IVR has been shown to effectively improve symptoms of ideomotor apraxia [26], induce positive changes in inflammation levels, oxidative stress, and BDNF serum biomarkers, and enhance functional evaluation scores in individuals with chronic stroke [29]. IVR appears to be particularly beneficial for individuals with mild to moderate impairments of the upper limbs.

IVR contributes to cortical reorganization, positively influencing motor function, bilateral primary motor cortex connectivity, and improvements in apraxia. Furthermore, IVR has been associated with enhanced scores on the UEFMA, increased Wrist AROM, and a more engaging rehabilitation experience.

However, the reviewed studies exhibit several limitations, including small sample sizes, which may affect the generalizability of the findings. Moreover, variability in the control of standard therapy protocols across study populations introduces potential bias into the results.

To strengthen the evidence and validate these outcomes, large-scale, rigorously designed studies are required.

### 4.2. Chronic Stroke

In individuals with chronic stroke, upper-limb (UL) motor impairments persist in most cases, significantly limiting the ability to perform BADLs. The effectiveness of IVR in improving motor skills in this population has shown mixed results [14,28]. Weber argues that the motor performance improvements observed in his study did not reach statistical significance, likely due to the small sample size, the severity of impairment within the studied population, and possibly insufficient treatment intensity. A larger study may yield statistically significant results. In contrast, Erhardsson’s study, despite a smaller sample size, showed that all participants demonstrated improvements in upper-extremity activity capacity, regardless of the severity of their impairment. Those receiving the highest training dose showed progress across multiple outcome measures. Furthermore, according to Mullick and collaborators, these patients exhibited fewer improvements compared to healthy controls [27].

IVR has been employed to assist in reaching objects while avoiding obstacles in both single and dual-task scenarios. A study comparing 13 individuals with mild stroke to 11 healthy controls found that the stroke group scored lower in both task execution and speed. The study also revealed a link between confidence in arm ability and success in task performance. IVR may enhance confidence through its graded difficulty levels and reward structure. Moreover, incorporating real-life scenarios may further boost the patient’s confidence in using the affected limb in everyday settings.

Weber and collaborators applied mirroring strategies in IVR to 10 chronic stroke patients with upper-limb hemiparesis [28]. The intervention consisted of 12 sessions, each lasting 30 min. Unlike Mekbib’s study [25], improvements in motor function, as measured by the Fugl–Meyer Upper-Extremity (FM-UE) and Action Research Arm Test (ARAT) scales, were not statistically significant. Key limiting factors included a small sample size and the severity of impairment. Another important factor was the insufficient intensity of the training, which should have been higher due to the low neuroplasticity observed in stroke patients during the chronic phase. When compared to traditional physical therapy, no significant difference in limb improvement was observed, although patients with less severe impairment showed better results [30,31]. Huang and collaborators demonstrated more substantial changes in serum inflammatory, oxidative stress, and neurotrophic biomarkers in the IVR group compared to the occupational therapy group. Consistent with previous studies, the IVR group also showed more significant improvements in upper limb functional evaluation and active range of motion. Longer studies are needed to further analyze the impact of IVR on serum biomarkers in the chronic phase, as well as larger sample sizes to better identify suitable candidates for this type of training [31]. According to Schuster-Amft and collaborators, IVR training offers multiple advantages and positive effects for upper limb motor recovery when compared to traditional training [27]. In this study, chronic stroke patients (at least six months post-stroke) with mild to severe impairments in upper limb movements were evaluated. While no significant differences were reported between the groups, the most notable improvements occurred during the first two weeks, after which progress stabilized until the final assessment. One advantage of IVR is its ability to customize characteristics and activities. However, Erhardsson et al.’s research focused on commercially available, ready-to-use systems for upper limb motor recovery in chronic stroke patients. While these systems are inexpensive and engaging, they lack the flexibility to be tailored to individual needs. Participants in this study were able to choose two games from five available options and select the duration of each session. The study found that the optimal amount of training time for significant upper limb improvement was 900 min (at least 30 min, three times per week, for 10 weeks). While IVR technology shows promise, several limitations remain, including small sample sizes [28,31], a lack of previous studies [30], and only being suitable for mild stroke patients [27]. Moreover, it is necessary to provide an IVR training expert [14]. Therefore, IVR plays a significant role in post-stroke functional recovery. It has demonstrated positive effects in both acute and chronic stroke stages, contributing to improvements in motor functionality, strength, dexterity, and range of motion [14,25,26,27]. It promotes neuroplasticity [29] and implements the capacity to schedule a movement [39].

Another review [38] also highlighted that VR protocols may offer new opportunities for stroke rehabilitation, but also emphasized the need for more extensive trials. IVR demonstrates similar effects across different groups, but with fewer compromises and better results compared to traditional methods. The Action Research Arm Test indicates that participants who receive more training achieve superior outcomes. Some improvements, such as enhancements in sensory function or upper limb muscle activity, were not statistically significant. In the IVR groups, an increase in limb function was observed, while the standard therapy group showed improvements in intrinsic sensory function. Positive results also support the use of IVR for biomarker improvements, as reflected in the FMA-UE, AROM, and Rating of Perceived Exertion (RPE) metrics, with an average increase of 12 points.

However, the studies reviewed present several methodological limitations, including small sample sizes and insufficient study duration to fully assess long-term effects. Furthermore, the lack of prior studies incorporating electroencephalogram (EEG) measurements represents another obstacle to a complete understanding of the collected data. Additionally, the consistent presence of a rehabilitation specialist specializing in virtual reality games during all sessions may have influenced the results, introducing a supervision variable that is not replicable in real-world conditions.

IVR holds significant potential for chronic stroke rehabilitation by innovating therapeutic approaches through enhanced integration into treatment pathways, greater personalization of interventions, and broader applications. As costs decrease, the technology will become more accessible, allowing its use even in home settings. However, large-scale studies are necessary to validate its benefits and establish standardized protocols.

### 4.3. Parkinson

Currently, the approach to PD is multidisciplinary, combining pharmacological and surgical interventions with physical therapy and adapted physical activity [40]. The integration of IVR into traditional therapy has gained relevance in cognitive and motor rehabilitation for patients with neurological disorders such as PD. This technological advancement has led to the development of the virtual Box and Block Test (VR-BBT), a reliable tool for measuring manual dexterity. Oña and collaborators demonstrated that the VR-BBT could be used as a clinical assessment to measure upper limb manual dexterity in patients in the early stages of PD [13]. Other studies have reported more significant improvements in upper limb movements, while patients also experienced enjoyment and satisfaction [16] even when compared to non-immersive VR. However, IVR has also been associated with more execution errors and greater pressure to perform well compared to traditional methods [40]. Sànchez Herrera-Baeza et al. showed greater improvements in hand grip, fine coordination, and gross dexterity in speed movements of the impaired limb, without any adverse effects. The CSQ-8 questionnaire on customer satisfaction yielded high scores, reflecting 100% compliance with the therapy [16]. One limitation of these studies is the small sample size [40], and it is also necessary to involve an IVR and PD training expert, as this technology is not suitable for every PD patient [16,17].

In conclusion, IVR is safe, effective, and feasible for PD rehabilitation. It serves as both a therapeutic tool and a clinical test for measuring upper-limb dexterity [16,40]. IVR sessions help improve hand grip, fine coordination, and gross motor skills [16]. Another review supports these findings, recognizing the potential of IVR in this field and highlighting its feasibility, usability, and safety, with promising benefits in addressing the common symptoms experienced by PD patients [34].

IVR proved to be enjoyable and more effective in managing time and tremors in the UPDRS test compared to the non-immersive VR group, which made fewer errors. Both groups showed improvements in the BBT. Significant progress was noted in strength, fine motor skills, gross coordination, and increased movement speed on the affected side. Participants reported high satisfaction, although the experience posed a mental challenge.

The studies presented several methodological limitations, including a small sample size, which limits the generalizability of the results to the broader PD population. Additionally, fatigue necessitated shorter breaks, which could have affected the therapy’s effectiveness. The research focused only on patients with mild to moderate PD, excluding those with more advanced forms of the disease. Finally, continuous professional monitoring was required throughout the study.

IVR shows promise in improving motor function, reducing tremors, and enhancing coordination. Moreover, personalized rehabilitation protocols could optimize recovery for each individual patient.

### 4.4. Amputation

Videogames provide valuable support to traditional physical therapy. Their engaging and enjoyable nature enhances motivation to engage in physical activity, replacing repetitive and monotonous exercises. The use of video games has been shown to produce positive effects across motor, cognitive, and emotional domains [19]. The application of IVR in individuals with amputation has demonstrated benefits in muscle strength, prosthetic limb control, and self-perceived competence, all with a high level of engagement. During IVR activities, participants reported low psychological pressure and tension [19,41]. The study also revealed that visual feedback could modulate pain perception [41]. Among various assistive tools, myoelectric prostheses are notable for improving the sensation of control, while other devices, such as mirror-box therapy (MBT), rely on the reflected image of the unaffected arm. However, MBT has certain limitations that may reduce its effectiveness, which IVR may help mitigate [41]. A questionnaire assessing efficacy indicated that two out of three participants experienced a greater sensation of control compared to following standard methods, with one participant stating, “I perceived the illusion of having both hands during the entire experiment” [41]. Despite promising results, limitations such as small sample sizes and insufficient studies on this topic need to be considered [19,41].

Individuals with amputation who utilized IVR alongside traditional therapy achieved similar outcomes to those observed in PD rehabilitation [19,41]. Furthermore, IVR was found to be effective in alleviating phantom limb pain [41]. While the results are encouraging, most studies remain at the case series level, which warrants caution when applying clinical recommendations [37].

All participants showed improvements in muscle strength and coordination. They also reported high satisfaction, including increased perceived competence, freedom of choice, and usefulness, while experiencing low levels of pressure and tension. Additionally, they exhibited improved control of the amputated limb, suggesting potential benefits for neuro-motor rehabilitation.

The study had several methodological limitations, including a small sample size and a lack of quantitative testing, which undermine the robustness of the findings. In one case, a less engaging game lowered participants’ motivation, potentially affecting the intervention’s effectiveness. Additionally, research on amputation and IVR remains limited, with only two articles available, which restricts the depth of analysis.

To improve the validity of future studies, increasing the sample size will be crucial. Additionally, activities should be designed to be more engaging in order to enhance motivation and reduce dropout rates, thus maximizing the potential benefits of IVR.

### 4.5. Fibromyalgia

Two out of three individuals with FS experience a condition known as kinesiophobia, characterized by a fear of exercise and movement. This condition can lead to avoidance behaviors, particularly in patients with maladaptive tendencies, and may also result in the nocebo effect. The nocebo effect occurs when negative expectations about a treatment result in poorer outcomes. Inactivity due to kinesiophobia leads to the loss of muscle strength, endurance, mobility, and functionality, which in turn exacerbates mental health issues and pain symptoms [36]. IVR has been shown to provide significant pain relief, particularly in chronic and acute pain conditions. Research indicates that pain relief is more substantial with immersive VR, which offers greater interactivity, resolution, and tracking accuracy [35]. According to Hoolahan and collaborators (2019), IVR is beneficial in managing both pain and kinesiophobia, offering more motivation compared to traditional physical therapy [38]. A randomized study by Gulsen et al. confirmed Hoolahan’s findings, demonstrating similar pain and kinesiophobia improvements with IVR, with no adverse side effects [34]. Tuck et al.’s randomized study also supports these findings, showing that IVR results in improvements similar to traditional therapy, but with additional psychological benefits and increased enjoyment [37]. Darnall’s study (2020) revealed that IVR significantly improves pain intensity, mood, sleep quality, and stress levels compared to audio-only devices [36]. Furthermore, Christensen et al. (2023) found that IVR enhances pain threshold and reduces pain catastrophization, even in healthy subjects [35].

IVR has also shown promise in the treatment of FS, providing relief from constant pain and distracting patients from discomfort [32,33,42,43,44]. It gradually helps restore functionality through movement, empowering patients to rediscover their abilities and potential [32,43,44].

IVR can be used remotely under the guidance of medical professionals, offering a convenient tool for home-based therapy.

In summary, IVR has demonstrated positive effects in individuals with upper-limb issues due to stroke, PD, amputation, and pain conditions such as FS. However, not all patient populations are suitable candidates for this therapy, as the results are highly dependent on the severity of the condition and the presence of comorbidities. Moving forward, the development of guidelines for IVR-based physical activity protocols could significantly enhance the independence of impaired individuals and reduce chronic disabilities.

Despite its promising benefits, existing studies present several methodological limitations. The small sample sizes, gender bias (as most studies involved women), and the lack of control groups limit the generalizability of the findings. Additionally, the pain assessed in these studies was experimental rather than clinical, and analgesic medications were not evaluated. Pain was self-reported by participants, which may have introduced bias. The absence of a control group also raises concerns about the potential placebo effect, which could undermine the validity of the results.

IVR holds promise as a tool for pain management through therapeutic exergames, particularly when applied in home-based settings. It has the potential to improve movement perception, reduce pain intensity, and promote relaxation. When integrated with multidisciplinary therapies, IVR could contribute to both motor function rehabilitation and improved mental health. Further studies are needed to address the identified limitations and refine the use of IVR in clinical practice.

## 5. Conclusions

This review delves into the use of IVR in upper-limb rehabilitation, specifically for patients with stroke, PD, MS, amputation, and FS. By synthesizing recent literature, the review highlights both the potential benefits and key challenges of IVR in these contexts. While substantial research exists on non-immersive and semi-immersive virtual reality, such as the work of Masmoudi and colleagues [45], the field of truly immersive virtual reality remains underdeveloped and poorly understood, particularly in relation to stroke, PD, amputation, and FS.

IVR serves as a valuable tool in physical therapy for various pathologies, particularly in the rehabilitation of upper limb function. It provides motivating training that targets specific goals, offering engaging and interactive experiences designed to enhance patient involvement and drive progress in motor recovery [39]. The combination of entertainment, fun, music, and quick feedback on scores plays a significant role in boosting motivation. By incorporating prizes and clear objectives, IVR not only keeps patients engaged but also enhances their involvement in the rehabilitation process, making therapy sessions more enjoyable and less repetitive [19]. The virtual environment enables users to simulate real-life scenarios (e.g., crossing the road), thereby enhancing autonomy and independence. It also offers diverse scenarios that provide comfort while minimizing distractions from external stimuli [11,18,19].

This approach has been proven to be a safe, practical, user-friendly tool, suitable for individuals of all ages, and customizable to meet individual needs.

IVR has shown promising potential across various medical conditions, including both acute and chronic stroke, PD, amputation, and FS. While each condition presents distinct challenges, IVR consistently yields benefits in motor rehabilitation, neuroplasticity promotion, and pain management.

For stroke patients, IVR facilitates motor recovery by promoting neuroplasticity and improving functional outcomes, particularly during the acute and subacute phases. However, limitations such as small sample sizes and the need for tailored training protocols underscore the need for further investigation. In the case of chronic stroke, IVR can enhance upper limb function, self-confidence, and patient engagement, although its effectiveness varies depending on the severity of impairment and the intensity of training.

IVR also serves as both a training tool and an assessment method for manual dexterity in PD. Research indicates improvements in hand grip, coordination, and movement speed, although concerns about execution errors and the need for expert supervision persist. Likewise, in individuals with amputations, IVR has been shown to improve prosthetic control, muscle strength, and self-perceived competence, while also offering psychological benefits, such as reduced anxiety and increased motivation.

Patients with FS benefit from significant pain relief and the improved management of kinesiophobia through IVR. The immersive nature of IVR effectively diverts attention from pain while facilitating functional recovery without adverse effects. Furthermore, IVR’s adaptability enables remote supervision, enhancing accessibility and patient compliance. Additional benefits include high levels of embodiment and engagement during activities, which may further boost patient compliance. Given that newer immersive virtual reality headsets are increasingly accessible in terms of usability and cost, the home use of this technology could optimize healthcare treatments. This would substantially increase the volume of therapy sessions without adding additional workload to medical facilities and healthcare professionals.

In summary, IVR has proven beneficial for individuals with upper-limb impairments resulting from stroke, PD, amputation, and FS. However, not all populations are suited for this technology, as the effectiveness of IVR depends on the severity of the condition and the presence of comorbidities. The results of these studies suggest that, with appropriate guidelines, an adapted protocol for physical activity using IVR could help improve the independence of affected individuals and reduce chronic disabilities.

Despite its advantages, IVR research faces challenges, including small sample sizes, a lack of standardized protocols, and the integration of IVR specialists into rehabilitation teams. Further large-scale studies are crucial to optimize the application of IVR, tailor interventions to individual needs, and validate its long-term benefits, as emphasized by Diriba Kenea and colleagues [46]. Additionally, several concerns must be addressed, including the risk of cybersickness [47] and the potential negative impact of impaired visual and perceptual capabilities in elderly patients [48]. Larger-scale studies are necessary to better understand the full impact of this technology on diverse populations. Nevertheless, IVR represents a promising, engaging, and effective tool in modern rehabilitation, complementing traditional therapies and enhancing patients’ quality of life.

## Figures and Tables

**Table 1:** Picost scheme.

PICOST		Questions	Area of Interest	Search Terms	Exclusion Criteria
P	patient population problem	how would I describe a group of patients similar to mine?	≥18 years old	“adult” or “elder”	<18 years old
I	intervention prognostic factor or exposure	which main intervention, prognostic factor or exposure am I considering?	motor rehabilitation with virtual reality. Dysfunctions of the upper limb.	(“re-education” or “rehabilitation” or “physical activity”) and (“virtual reality” or “vr” or “immersive virtual reality” or “HMD” or “head-mounted display” or “IVR”) and “upper limb” or fibromyalgia syndrome	non-immersive virtual reality
C	comparison or intervention	what is the main alternative to compare with the intervention?	no intervention		
O	outcome you would like to measure or achieve	what can I hope to achieve, measure, influence?	1—improvement of residual capacities and recovery of lost or damaged motor functions		
			2—perceived quality of life	“Quality of life” or “QoL”	
			3—activities of daily living	“ADL” or “activity of daily living”	
S	study types	what is the best type/design?	RCT’s experimental analytical studies case report		
T	time	are there any time restrictions?		filter: 2018–2023	

**Table 2:** Acute and subacute stroke articles included.

Article	Objective	Subjects	Tool	Duration	Activity	Outcome	Results	Limits
Patel et al. (2019) [24]	testing whether +8 h of intensive training with IVR improves impairment and changes in cortical reorganization compared to usual therapy	13 subjects 30–80 years old	IVR + usual therapy vs. t. usual	8 sessions of 1 h intensive training (200–300 movements) + 3 h usual therapy	hand activities	UEFMA; Wrist AROM; WMFT; surface EMG	changes in both groups in cortical reorganization. IVR results in better UEFMA and Wrist AROM of the pulse and in the amusement scale. Control group less tension.	small sample
Mekbib et al. (2020) [25]	check cortical and physical changes with unilat. and bilat. mirroring ex. with IVR	8 subacute stroke and 13 healthy controls	IVR	1 h VR and 1 h conventional therapy per day, 4 days/week for 2 weeks	catching and moving a ball with unilateral and bilateral mirroring es	FMA-UE; MRI	improvements in motor function and bilateral M1 connectivity	small population lack of control with usual therapy
Park et al. (2021) [26]	check whether IVR improves apraxic symptoms	1 acute subject 56 years old	IVR vs. VR vs. AR vs. T.O.	20 min a day, 5 days a week, for 4 weeks	reaching and grasping; consecutive grasping and releasing gestures	MMSE; UEFMA Modified Barthel Index; upper limb apraxia score test	improvements in apraxia with IVR and consecutive grasp and release gestures	large-scale studies needed

**Table 3:** Chronic stroke articles included.

Article	Objective	Subjects	Tool	Duration	Activity	Outcome	Results	Limits
Schuster-Amft et al. (2018) [27]	the aim of this study was to compare virtual reality-based training with conventional therapy	54 subjects	IVR vs. conventional therapy	16 sessions, 45 min, 4 weeks	e.g., reaching, grasping, releasing. With Bi-Manua Trainer	BBT; CAHAI-13; SIS	similar effects between groups. Better IVR group	number of patients in each group
Erhardsson et al. (2020) [14]	exploring the potential of virtual reality for chronic stroke rehabilitation	7 subjects	IVR	10 weeks between 200 and 900 min	5 commercial games	ARAT; BBT; questionnaire ABILHAND; FMA-UE	improvement of the Action Research Arm Test participants with more training has higher results	have a researcher, an expert in rehabilitation and VR games, on site for all training sessions
Weber et al. (2019) [28]	using IVR for mirror therapy	10 subjects 25–68 years	IVR	12 sessions of 3 treatment blocks for 5 min performed twice for a total of 30 min per session	1st block: global limb movements 2nd block: lifting and moving rocks 3rd block: daily activities	FMA-UE; ARAT	non-significant improvement	small sample, relatively severely impaired subjects and insufficient intensity
Song et al. (2021) [29]	to determine the effect of an intervention tool combining an immersive VR system with bilateral upper limb training on EEG measurements in stroke patients with chronic hemiplegia	12 subjects with hemiplegia	IVR bilateral arm training vs. normal bilateral	5 times a week, 4 weeks, 30 min	daily activities, such as switching on lights, organizing a chest of drawers, organizing a kitchen, watering plants and buying items in a convenience store	UL function EMG; MFT; sensory function testing of the upper limb	increase in limb function in the IVR group, non-significant improvement in sensory function test, improvement in intrinsic sensory function in the standard group. No significant improvement in superior limb muscle activity	small sample, lack of prior studies with ECG
Mullick et al. (2021) [30]	identifying whether and to what extent cognitive–motor deficits in well-healed stroke individuals affect the ability to adapt	13 strokes 63.9 ± 8.1 years 11 healthy 63.7 ± 10.9 years)	IVR	4 experimental blocks consisting of 15, 60, 15 and 60 exercises, respectively. Rest 2 to 5 min between	reaching a bottle with obstacle avoidance in single and dual tasks with memorization activities	FMA-UE; elbow flexor spasticity CSI; WMFT; MAL; CAHM	better results in healthy subjects and positive correlation between confidence in arm strength and exercise success	results are limited to subjects with chronic mild stroke. The sample size was too small
Huang et al. (2022) [31]	to investigate the effects of VRT on serum markers of inflammation, oxidative stress and neuroplasticity, and on upper limb motor function in chronic stroke patients	30 subjects	IVR vs. conventional occupational therapy	16 sessions, 60 min, 2/3 sessions per week	commercial games	levels of: heme oxygenase 1, 8-hydroxy-2-deoxyguanosine, brain-derived neurotrophic factor, interleukin-6; FMA-UE; AROM, ARAT, RPE	positive results supporting IVR for biomarkers and FMA-EU and AROM and RPE by an average of 12	subjects only with chronic stroke, lack of differences with subj. healthy, period too short, small sample

**Table 4:** Parkinson’s disease articles included.

Article	Objective	Subjects	Tool	Duration	Activity	Outcome	Results	Limits
Cikajlo et al. (2019) [32]	study the functional improvements, motivational aspects and clinical effectiveness of IVR compared to non-immersive VR	20 subjects	IVR vs. VR non-immersive	10 sessions 3 weeks	grasping, moving the object and releasing	modified IMI; BBT; UPDRS	IVR better in handling time, tremor in UPDRS test and fun. The laptop group had fewer errors and less pressure/voltage. Both improved BBT	small sample
Sánchez-Herrera-Baeza et al. (2020) [16]	assessing quantitative and qualitative effects in IVR treatment	6 subjects 69–80 years old	IVR	30 min, 3 times a week, 6 weeks	upper limb function exercises + cognitive exercises	Jamar hydraulic hand dynamometer; BBT; PPT; ARAT; CSQ-8	significant improvements in strength, fine movement and coarse co-ordination dexterity, and speed movements on the affected side, and high satisfaction but a mental challenge	fatigue reduced pause, professional monitoring required, small sample, cannot be generalized to all subjects with PD
Oña et al. (2020) [17]	to evaluate the validity, feasibility and psychometric properties of a fully immersive VR-BBT to assess manual dexterity in PD patients	20 subjects mean age 74.38 ± 0.94 years	IVR BBT vs. Real BBT	3 trials	shifting of cubes	physical BBT; virtual BBT; satisfaction questionnaire	correlation between VR-BBT and BBT; correlation between VR-BBT score with PD severity as measured by the Hoehn and Yahr scale	the sample included only patients with mild to moderate stage PD

**Table 5:** Amputation articles included.

Article	Objective	Subjects	Tool	Duration	Activity	Outcome	Results	Limits
Hashim et al. (2021) [19]	examining the impact of IVR in muscle training, coordination and motivation	5 amputees, 5 able-bodied subjects	IVR	4 weeks, 10 sessions, 1 h	games: Crate Whacker, Race the Sun, Fruit Ninja e Kaiju Carnage	physical BBT; virtual BBT; EMG; IMI	increased muscle strength and coordination for all. High scores for interest, perceived competence, choice and usefulness, but low for pressure and tension	an uninspiring game lowered motivation to finish it
Henriksen et al. (2017) [33]	creation of the illusion of the reacquisition of a limb	3 amputees with phantom limb pain	IVR + electrostimulation	15 sessions in 5 weeks of 60/90 min	1 bending game 2 frequency discrimination game 3 position discrimination game	7-point Likert scale questionnaire	two participants increased control of the amputated limb	lack of quantitative tests and small sample

**Table 6:** Fibromyalgia articles included.

Article	Objective	Subjects	Tool	Duration	Activity	Outcome	Results	Limits
Gulsen et al., 2022 [34]	evaluate effects of VR combined with exercise training in fibromyalgia patients	20 fibromyalgic women (age 18–65)	IVR simulation	2 sessions per week, 20’ each, for 8 weeks	football game (countering balls from different directions) + dungeon game (tilting the trunk to avoid guillotines)	pain VAS; MSOT; Tampa scale for kinesiophobia; FIQ; FSS	IVR had positive effects in reducing pain, kinesiophobia, fatigue and improving emotional aspect of life quality	only women included, small sample
Christensen et al., 2023 [35]	investigate VR effects on cold pain threshold, tolerability, intensity in fibromyalgia patients and pain-free subjects; explore correlations between VR and pain catastrophization	22 fibromyalgic women + 22 healthy women (average 47,6)	IVR simulation	one session, 50’ with 20’ rest	birthday party simulation (while dominant foot is placed into ice water tub)	pain VAS, cold pain tolerance, PCS	IVR had positive effects in pain threshold of pain-free subjects but not among fibromyalgia patients. No correlation has been found regarding pain catastrophyzation	only women included, small sample, experimental pain and not clinic pain
Darnall et al., 2020 [36]	evaluate feasibility and efficacy of a self-administered VR program for chronic pain, compare the VR treatment with an audio-only treatment	97 fibromyalgia and/or chronic pain patients (age 18–75)	IVR simulation	12 to 24 sessions, 15’ each, in 21 days	visual biofeedback that amplifies the environment responding to the users’ physiological behavior during breathing exercises	DVPRS; average Pain Intensity 11-points scale; PCS; PGIC	IVR had positive effects in reducing pain intensity, stress and improving mood of participants; better than audio only	no pain meds are evaluated in this study, pain is self-measured
Tuck et al., 2022 [37]	test efficacy of VR in a chronic pain treatment center and assess the acceptability of an active VR treatment program	29 fibromyalgia and/or chronic pain patients (age 18–70)	IVR simulation	2 sessions per week for 6 weeks	commercially available games encouraging full-body movements (Fruit Ninja, Holodance et al.)	BPI; Tampa scale for kinesiophobia; PGIC	IVR had positive effects in reducing pain intensity, improving scores and fun in patients	no control group, possible placebo effect
Hoolahan et al., 2019 [38]	evaluate if VR can be used as physical activity approach in fibromyalgia patients	8 adults	IVR simulation	2 sessions, 5’ each	picking up, throwing and dodging snowballs	RPE, satisfaction questionnaire	IVR was perceived as motivating, reducing perceived effort. All subjects except one wanted to continue playing	no control group, possible placebo effect

## Data Availability

Data are fully available upon request.

## Abbreviations

The following abbreviations are used in this manuscript:

ARAT	Action Research Arm Test
AROM	Active Range Of Motion
BADL	Basic Activities of Daily Living
BBT	Box and Block Test
BPI	Brief Pain Inventory
CAHAI-13	Chedoke–McMaster and Hand activity Inventory
CAHM	Confidence in Arm and Hand Movement scale
CSI	Composite Spasticity Index
CSQ-8	Customer satisfaction questionnaire
DVPRS	The Defense and Veterans Pain Rating Scale
FIQ	Fibromyalgia Impact Questionnaire
FMA-UE	Fugl-Meyer Assessment for the Upper Extremity
FS	Fibromyalgia Syndrome
FSS	Fatigue Severity Scale
IMI	Intrinsic Motivation Inventory
IVR	Immersive Virtual Reality
MDPI	Multidisciplinary Digital Publishing Institute
MFT	Manual Function Test
MMSE	Mini-Mental State Examination
MAL	Motor Activity Log
MS	Multiple Sclerosis
MSOT	Modified Sensory Organization Test
PCS	Pain Catastrophizing Scale
PD	Parkinson Disease
PGIC	Patient Global Impression of Change scale
PICOST	Population, Intervention, Comparator, Outcome, Study design, and Timeframe
PPT	Purdue pegboard coordination test
PRISMA	Preferred Reporting Items for Systematic Review and Meta-Analysis
RPE	Rating of Perceived Exertion
SIS	Stroke Impact Scale
UEFMA	Upper Extremity Fugl–Meyer Assessment
UL	Upper Limb
VAS	Visual Analogic Scale
VR	Virtual Reality
WMFT	Wolf Motor Function Test
